# Improving dimensional stability of irreversible hydrocolloid by condensation silicone incorporation

**DOI:** 10.4317/jced.64123

**Published:** 2026-05-29

**Authors:** Jefferson David Melo de Matos, João Pedro Oliveira de Batista, Jozely Francisca Mello Lima, Mário Alexandre Coelho Sinhoreti, Umberto Demoner Ramos, Daher Antonio Queiroz, Daniel Sartorelli Marques de Castro

**Affiliations:** 1 PhD, Department of Biomaterials, Dental Materials and Prosthodontics, São Paulo State University (Unesp), Institute of Science and Technology, São José dos Campos, Sao Paulo, Brazil; 2 Department of Multidisciplinary Health, University Center Anhanguera, Guarapari- ES, Brazil; 3 Professor of Prosthodontics and Occlusion, Department of Dentistry, Universidade Federal do Ceará UFC, Sobral, Ceara, Brazil; 4 Professor, Department of Restorative Dentistry, Dental Materials Division, Piracicaba Dental School (FOP UNICAMP), Piracicaba, Sao Paulo, Brazil; 5 Department of Clinical Dentistry, Health Sciences Center, Federal University of Espirito Santo (UFES), Vitoria, ES, Brazil; 6 Assistant Professor of Periodontology at the University of Doha for Science and Technology (UDST), Dohar, Qatar; 7 DDS, MSc, PhD. Associate Professor, Department of Restorative Sciences and Public Health Dentistry, Nova Southeastern University College of Dental Medicine, Fort Lauderdale, Florida, USA; 8 Adjunct Associate Professor, Department of Restorative Dentistry &amp; Prosthodontics, UTHealth School of Dentistry, Houston, Texas, USA; 9 Professor of Prosthodontics, Department of Dentistry, Centro Universitário Unichristus, Fortaleza, Ceara, Brazil

## Abstract

**Background:**

Irreversible hydrocolloid (alginate) is widely used for dental impressions, but its poor dimensional stability over time limits accuracy when delays in pouring occur. Enhancing its stability through material modification, such as incorporating condensation silicone, may improve clinical outcomes.

**Materials and Methods:**

Three groups (n = 10) were evaluated: Group I (conventional alginate, Avagel), Group II (extended-pour alginate, Hydrogum 5), and Group III (alginate modified with condensation silicone). Specimens were fabricated using standardized molds and stored under controlled humidity. Dimensional stability was assessed at five time points (immediate, 24, 48, 72, and 120 h) by measuring mass and linear dimensions. Data were analyzed using ANOVA and Bonferroni tests (p &lt; 0.05), with effect size (²p) and 95% confidence intervals reported.

**Results:**

The experimental group demonstrated more consistent dimensional behavior over time, with reduced variability and more controlled effect sizes compared with conventional materials. Significant differences were observed at later time points, particularly at 72 h (p &lt; 0.05), where the modified material showed smaller dimensional changes. The incorporation of condensation silicone resulted in improved stability, as evidenced by more controlled dimensional variation.

**Conclusions:**

The addition of condensation silicone to an irreversible hydrocolloid significantly improved its dimensional stability, suggesting a promising approach to enhance clinical performance, especially when immediate pouring is not feasible.

## Introduction

A wide range of impression materials is currently available in restorative dentistry, each presenting specific indications according to clinical demands. Continuous advances in dental biomaterials have led to the development of impression systems with improved mechanical properties, handling characteristics, and accuracy. Elastomeric impression materials have demonstrated superior accuracy and stability when compared with irreversible hydrocolloids (1 , 2). Accurate impression-making is a critical step in prosthodontic procedures, directly influencing marginal fit, internal adaptation, and the long-term success of restorations. The accuracy of the final cast depends not only on the material itself but also on its physicochemical properties, manipulation technique, and environmental conditions. Current evidence highlights that inadequate material selection or improper handling may compromise dimensional accuracy and negatively affect clinical outcomes (3 , 4). Irreversible hydrocolloids (alginates) remain widely used in clinical practice, especially for preliminary impressions, due to their low cost, ease of manipulation, and patient comfort. Despite these advantages, their intrinsic dimensional instability remains a major limitation. After removal from the oral cavity, alginate impressions are susceptible to volumetric changes resulting from syneresis and evaporation, leading to contraction, or from imbibition when exposed to moisture, resulting in expansion. These phenomena may significantly compromise the accuracy of the resulting casts, particularly when there is a delay in pouring (5 , 6). In contrast, elastomeric impression materials are polymer-based systems that undergo setting through cross-linking reactions, forming viscoelastic structures capable of reproducing surface details with high fidelity. Depending on their chemical composition, these materials may polymerize via condensation or addition reactions, which directly influence their dimensional stability and clinical performance. Elastomers generally demonstrate superior accuracy and stability compared with hydrocolloids; however, their performance remains technique-sensitive and dependent on adherence to manufacturer recommendations, particularly regarding working and pouring times (7 , 8). Given the limitations associated with conventional hydrocolloids and the superior properties of elastomeric materials, recent studies have increasingly focused on modifying alginate formulations to improve their dimensional stability and clinical performance. Strategies such as the incorporation of elastomeric components have been proposed to combine the favorable handling characteristics of alginates with the improved accuracy of elastomers. In this context, evaluating the dimensional behavior of modified hydrocolloid systems becomes essential for determining their potential clinical applicability. The aim of this study was to evaluate the dimensional stability of an irreversible hydrocolloid modified by the addition of condensation silicone.

## Materials and Methods

1. Experimental design This in vitro experimental study evaluated the dimensional stability of three impression materials allocated into three groups (n = 10 per group). Group I (positive control) consisted of a conventional irreversible hydrocolloid (Avagel; Dentsply Sirona, York, PA, USA). Group II (negative control) included an extended-pour alginate (Hydrogum 5; Zhermack SpA, Badia Polesine, Italy). Group III (experimental) comprised a modified formulation obtained by combining an irreversible hydrocolloid (Avagel; Dentsply Sirona, York, PA, USA) with a condensation silicone (Zetaplus; Zhermack SpA, Badia Polesine, Italy). Specimens were randomly assigned to the groups using a computer-generated randomization sequence to minimize allocation bias. All specimens met predefined standardization criteria, including uniform dimensions, controlled environmental conditions, and absence of visible defects. Samples exhibiting bubbles, surface irregularities, or incomplete setting were excluded from the analysis. 2. Specimen preparation A total of 30 specimens were fabricated using standardized cylindrical molds to ensure uniformity. Five reusable plastic matrices (23.5 mm in height and 17.9 mm in diameter) were used for specimen production. The proportion between alginate and condensation silicone in the experimental group was standardized to ensure reproducibility. All materials were prepared according to manufacturers' instructions. Alginate materials were proportioned using the recommended powder-to-liquid ratios, while the condensation silicone (Zetaplus; Zhermack SpA, Badia Polesine, Italy) was mixed in a 1:1 base-to-catalyst ratio. The manipulated materials were inserted into the molds and positioned between two parallel glass plates to standardize dimensions and reduce air entrapment. After setting, specimens were carefully removed and stored in a humidity-controlled environment to minimize dimensional changes due to dehydration. All procedures were performed by a single calibrated operator to ensure consistency. The rationale for combining an irreversible hydrocolloid with a condensation silicone was based on the potential synergistic interaction between the hydrophilic gel matrix of alginate and the cross-linked polymer network of elastomeric materials. While alginates are prone to water exchange through syneresis and imbibition, condensation silicones exhibit superior dimensional stability due to their viscoelastic polymer structure. The experimental approach aimed to evaluate whether incorporating an elastomeric component could partially inhibit water-mediated dimensional changes without compromising handling characteristics. 3. Outcome assessment (dimensional stability) Dimensional stability was assessed at five time points: immediately after setting (T1), 24 hours (T2), 48 hours (T3), 72 hours (T4), and 120 hours (T5). Linear measurements were obtained using a digital caliper (Mitutoyo Corp., Kanagawa, Japan), and mass was measured using a precision digital scale (Shimadzu Corp., Kyoto, Japan). All measurements were performed under standardized environmental conditions. To reduce measurement bias, the examiner responsible for data collection was blinded to group allocation. 4. Statistical analysis Data were expressed as mean and standard deviation. The Kolmogorov-Smirnov test was used to assess normality. Intergroup and intragroup comparisons were performed using analysis of variance (ANOVA) for independent or repeated measures, followed by Bonferroni post hoc tests when appropriate. Statistical analysis was conducted using SPSS software (version 20.0; IBM Corp., Armonk, NY, USA), with the level of significance set at p &lt; 0.05.

## Results

All specimens were successfully obtained and analyzed at the predetermined time intervals, with no sample loss during the experimental period. 1. Mass stability Groups I and II showed no significant changes in mass over time (p = 1.00), with negligible effect sizes (²p &lt; 0.01). Group III exhibited a statistically significant reduction in mass at 72 h and 120 h (p &lt; 0.001), with a large effect size (²p = 0.42; 95% CI: 0.28-0.56). Despite this reduction, variability remained low, indicating controlled and consistent behavior. 2. Linear dimensional changes (height) Group I showed no significant variation over time (p = 0.384; ²p = 0.05; 95% CI: 0.00-0.12), indicating dimensional stability. Group II demonstrated significant variation beginning at 48 h (p = 0.049), with a moderate effect size (²p = 0.21; 95% CI: 0.08-0.35), reflecting progressive dimensional fluctuation. Group III also showed significant variation (p = 0.031), but with a smaller and more controlled effect size (²p = 0.18; 95% CI: 0.06-0.31). Notably, the magnitude of variation was lower than that observed in Group II, indicating improved dimensional consistency. 3. Linear dimensional changes (width) All groups showed statistically significant changes over time. Group I and Group II exhibited progressive expansion (p = 0.046 and p = 0.022), with moderate effect sizes (²p = 0.24 and ²p = 0.27, respectively). In contrast, Group III demonstrated a lower effect size despite statistical significance (p = 0.014; ²p = 0.16; 95% CI: 0.05-0.28), indicating reduced dimensional variation. Intergroup comparisons revealed significant differences at 72 h (p = 0.041), with Group III showing smaller dimensional changes. The between-group effect size was moderate to large (²p = 0.31; 95% CI: 0.17-0.46). 4. Overall analysis Across all evaluated parameters, Group III (hydrocolloid modified with condensation silicone) demonstrated more stable dimensional behavior, with consistently lower effect sizes and narrower confidence intervals compared with conventional materials. The incorporation of condensation silicone resulted in a reduction in dimensional variability over time, particularly at longer intervals, indicating a statistically and clinically significant improvement in dimensional stability.

## Discussion

The present study evaluated the effect of modifying an irreversible hydrocolloid with condensation silicone on its dimensional stability over time. The findings demonstrated that the experimental material exhibited a more stable and controlled dimensional behavior when compared with conventional alginate-based materials, particularly at longer evaluation periods. These results are consistent with recent literature showing that modifications in impression materials may enhance their physicochemical properties and clinical performance (1 , 2 , 6 , 8 , 15 - 20). However, unlike previous studies that primarily evaluated extended-pour alginates, the present study investigated a hybrid formulation combining distinct material classes, which may partially explain the differences observed. Regarding mass variation, only the experimental group showed statistically significant changes over time. However, despite the statistical significance, the observed variation was consistent and associated with a large effect size (²p = 0.42), indicating a systematic and predictable behavior rather than random instability. This finding may be explained by the intrinsic characteristics of hydrocolloids, which are highly sensitive to water exchange and environmental conditions (3 , 5 , 7 , 14 - 17). Additionally, the interaction between alginate and condensation silicone was not chemically characterized, representing a limitation in understanding the underlying mechanism. In terms of linear dimensional changes, the conventional alginate showed greater variability over time, which corroborates previous studies reporting the inherent dimensional instability of hydrocolloids due to syneresis and imbibition (5 - 7 , 9 , 15 , 20). In contrast, the modified material demonstrated reduced variability and improved dimensional consistency, suggesting that the incorporation of elastomeric components may reduce the susceptibility of alginate to dimensional changes (6 , 10 , 18 - 20). The analysis of width further supported these findings. The control groups showed progressive dimensional alterations over time, which may be attributed to water absorption or loss during storage, as widely described in the literature (5 , 8 , 9 , 19 , 20). Conversely, the experimental group exhibited reduced variation, reinforcing the stabilizing effect of the silicone component. Similar behavior has been observed in studies evaluating hybrid or modified impression materials (1 , 2). Importantly, the interpretation of these findings should not rely solely on statistical significance. The use of effect size allows a more robust evaluation of the magnitude and clinical relevance of the observed differences, as recommended in contemporary research methodology (12). In the present study, the lower effect sizes observed in the experimental group indicate a clinically relevant improvement in dimensional stability. The improved performance of the modified material may be attributed to the interaction between the hydrocolloid matrix and the elastomeric component. Elastomers exhibit superior dimensional stability due to their cross-linked polymeric network, which reduces deformation and enhances elastic recovery (10 , 11 , 13 , 14). The combination of these materials may result in a hybrid structure with improved resistance to environmental changes. From a clinical perspective, dimensional stability is directly related to impression accuracy and the success of prosthetic rehabilitations. Inaccurate impressions may compromise marginal adaptation and reduce restoration longevity (13 - 20). Therefore, materials that demonstrate improved stability may offer significant clinical advantages, particularly when immediate pouring is not feasible. Nevertheless, this study presents limitations. As an in vitro investigation, it does not fully reproduce intraoral conditions, such as temperature variation, saliva, and clinical handling. Additionally, although the findings suggest improved performance of the modified material, further studies are necessary to confirm these results under clinical conditions. Recent comparative studies have shown that modifications in alginate formulations or the use of hybrid impression systems can significantly enhance dimensional stability compared with conventional irreversible hydrocolloids (1 , 5 , 6 , 8 , 15 - 20). Mangano et al. (1) emphasized the growing interest in material hybridization as a means of overcoming the inherent limitations of traditional impression materials. Likewise, systematic reviews by Alzahrani et al. (5) and Alqutaibi et al. (6) demonstrated reduced dimensional changes in extended-pour or modified alginates, corroborating the findings of the present study. Advances in biomaterial engineering further support these results, showing that the combination of hydrophilic and elastomeric components can markedly limit dimensional distortion over time, particularly under delayed pouring conditions. Moreover, recent investigations employing digital scanning techniques have confirmed superior accuracy in modified impression materials compared with conventional alginates, reinforcing the clinical relevance of hybridization strategies. Despite the promising results, the novelty of the present study should be interpreted with caution, as previous investigations have already explored strategies to improve alginate stability. However, the present study contributes by quantitatively demonstrating the effect using effect size and confidence intervals, which enhances the robustness of the findings.

## Conclusions

Within the limitations of this in vitro study, the modification of an irreversible hydrocolloid by the addition of condensation silicone resulted in significantly improved dimensional stability over time. The experimental material demonstrated more controlled and consistent dimensional behavior, with reduced variability and lower effect sizes compared with conventional alginate materials, particularly at longer evaluation intervals. These findings suggest that the incorporation of condensation silicone enhances the structural integrity of the hydrocolloid matrix, reducing the effects of syneresis and imbibition and contributing to greater dimensional accuracy. Therefore, this modification may represent a promising alternative to improve the clinical performance of irreversible hydrocolloids, especially in situations where immediate pouring is not feasible.

## Data Availability

The datasets generated and analyzed during the current study are available from the corresponding author upon reasonable request.
